# Physiotherapy utilisation and costs before lumbar spine surgery: a retrospective analysis of workers compensation claims in Australia

**DOI:** 10.1186/s12891-021-04129-4

**Published:** 2021-03-06

**Authors:** Joshua R. Zadro, Adriane M. Lewin, Priti Kharel, Justine Naylor, Christopher G. Maher, Ian A. Harris

**Affiliations:** 1 Institute for Musculoskeletal Health, The University of Sydney and Sydney Local Health District, Sydney, NSW Australia; 2 Ingham Institute for Applied Medical Research, South Western Sydney Clinical School, University of New South Wales, Sydney, NSW Australia; 3 Whitlam Orthopaedic Research Centre, Orthopaedic Department, Liverpool Hospital, Sydney, NSW Australia

**Keywords:** Rehabilitation, Fusion, Decompression, Physical therapy, Lumbar spine, Workplace

## Abstract

**Background:**

Understanding how much physiotherapy people receive before lumbar spine surgery could give insight into what people and clinicians consider an adequate trial of non-operative management. The aim of this study was to investigate physiotherapy utilisation and costs before lumbar spine surgery under a workers’ compensation claim in New South Wales (NSW), Australia.

**Methods:**

Using data from the NSW State Insurance Regulatory Authority, we audited physiotherapy billing codes used before surgery for people who received lumbar spine surgery from 2010 to 2018. We summarised, separately for fusion and decompression, the time from initiation of physiotherapy to surgery, number of physiotherapy sessions people received before surgery, total cost of physiotherapy before surgery, and time from injury date to initiation of physiotherapy. All analyses were descriptive.

**Results:**

We included 3070 people (800 had fusion, 2270 decompression). Mean age (standard deviation, SD) was similar between those who received fusion and decompression [42.9 (10.4) vs. 41.9 (11.6)]. Compared to people who had fusion, those who had decompression were more likely to not have any physiotherapy before surgery (28.4% vs. 15.4%), received physiotherapy for a shorter duration before surgery [median (interquartile range, IQR): 5 (3 to 11) vs. 15 (4–26) months], were less likely to have physiotherapy for ≥2 years before surgery (5.6% vs. 27.5%), had fewer physiotherapy sessions before surgery [mean (SD): 16 (21) vs. 28 (35) sessions], were less likely to have > 50 physiotherapy sessions before surgery (6.8% vs. 18.1%), and had lower total physiotherapy-related costs [mean (IQR): $1265 ($0–1808) vs. $2357 ($453–2947)]. Time from injury date to first physiotherapy session was similar between people who had fusion and decompression [median (IQR): 23 (9–66) vs.19 (7–53) days].

**Conclusions:**

There is variation in physiotherapy utilisation and costs before lumbar spine surgery for people funded by NSW Workers’ Compensation. Some people may not be receiving an adequate trial of physiotherapy before surgery, particularly before decompression surgery. Others may be receiving an excessive amount of physiotherapy before surgery, particularly before fusion.

**Supplementary Information:**

The online version contains supplementary material available at 10.1186/s12891-021-04129-4.

## Background

Low back pain (LBP) is the leading cause of years lived with disability worldwide and is responsible for a high economic burden [1]. In Australia, direct costs due to LBP are estimated at over AU$5 billion per year, with indirect costs estimated to be substantially more [2]. In the United Kingdom, the total annual cost of LBP is estimated to be ₤12 billion [3]. Although most people will experience some form of LBP in their lifetime [4], only a small percentage experience symptoms that limit their daily activity (7.3%) [5]. These people are thought to account for the majority of disability and costs associated with LBP [6, 7].

First-line care for acute LBP includes advice to stay active and reassurance of the favourable prognosis of most LBP cases, along with exercise and spinal manipulative therapy for those with persistent/chronic symptoms [8]. Clinical practice guidelines for LBP typically recommend against spinal fusion surgery and only recommend spinal decompression surgery for people with neurological symptoms consistent with radiological findings and when non-operative management has ‘failed’ [8, 9]. Nevertheless, rates of lumbar spine surgery are increasing globally. Between 2003 and 2013 in Australia, rates of decompression surgery for lumbar spine stenosis increased from 19.0 to 22.1 per 100,000 people, while rates of simple lumbar fusion and complex lumbar fusion increased from 1.3 to 2.8 per 100,000 and 0.6 to 2.4 per 100,000, respectively [10]. Rates of lumbar spine surgery are also increasing in the United States of America (USA) [11] and United Kingdom [12].

A key challenge in deciding whether a patient is suitable for surgery is determining what constitutes ‘failed’ non-operative management. Guidelines vary in their recommendations for how long people with LBP should receive non-operative management before considering surgery, ranging from 4 to 6 weeks to 2 years [9] . Physiotherapists are one of the key groups of health professionals involved in the non-operative management of LBP. Understanding how much physiotherapy people receive before lumbar spine surgery could give insight into what people and clinicians consider an adequate trial of non-operative management.

Several studies have investigated physiotherapy utilisation before lumbar spine surgery. Two retrospective cohort studies in the USA (*n* = 27,877 and *n* = 13,106 people, respectively) [13, 14] found 40% of people with symptomatic lumbar stenosis or spondylolisthesis had received physiotherapy or occupational therapy in the 2 years prior to lumbar fusion [13], and 16% with lumbar intervertebral disc herniation received physiotherapy or occupational therapy in the 3 months prior to lumbar microdiscectomy [14]. A cross-sectional survey of 229 people with LBP in Canada found half of the people received physiotherapy in the 2 years prior to having a consult for elective lumbar spine surgery [15]. Only one study, a retrospective analysis of 30,709 people in the United States, investigated the number of physiotherapy sessions people received before lumbar spine surgery [16]. They found people with lumbar disc herniation, on average, had 6 physiotherapy sessions in the 90 days before lumbar discectomy. All the above studies restricted the pre-surgery data collection period, presumably to specifically explore physiotherapy utilisation in a given time frame before surgery and to facilitate comparison with existing studies. Alternatively, it may be due to lack of complete data to capture the entire continuum from injury to physiotherapy to surgery. Restricting the data collection period to just prior to surgery however makes it difficult to determine how much physiotherapy people received – and for how long – from initiation of physiotherapy to surgery.

No study has investigated how much physiotherapy people receive – and for how long – from initiation of physiotherapy to lumbar fusion and lumbar decompression surgery, in Australia, or in a setting involving workers’ compensation. Filling these knowledge gaps will improve understanding of what health professionals and people consider ‘failed’ non-operative management or what they consider an appropriate amount of non-operative treatment before lumbar spine surgery. The primary aim of this study was to describe the time from initiation of physiotherapy to surgery in a cohort of people funded by workers’ compensation insurance in New South Wales (NSW), Australia, all of whom underwent lumbar fusion and/or lumbar decompression surgery. The secondary aims were to describe:

I. the number of physiotherapy sessions people receive before undergoing lumbar fusion and lumbar decompression surgery;
II. the total cost (per claim) of physiotherapy received before lumbar fusion and lumbar decompression surgery; and
III. the time from injury date to initiation of physiotherapy in people who later undergo lumbar fusion and lumbar decompression surgery.

## Materials and methods

### Data source

The NSW Workers’ Compensation system is the largest defined benefit system in Australia and compensates people who submit a claim for a work-place injury. Musculoskeletal pain caused by the demands of a job, as deemed by a health professional (e.g. general practitioner, physiotherapist), is classified as a work-place injury. In NSW, there were nearly 5000 work-place claims for back injuries/pain in 2015/16, at a cost of over $99 million and representing 20% of all major work-place injuries [17].

Most employers are required by law to have an insurance policy to cover the cost of workers’ compensation claims. Compensation mostly covers lost wages and use of health services but can also cover other needs (e.g. domestic assistance, education or training assistance, lump sum payment for permanent impairment). NSW Workers’ compensation is regulated by the State Insurance Regulatory Authority (SIRA). Insurers are required to provide SIRA with data on claimant characteristics (e.g. age, gender, occupation, workplace industry, date of injury, type of injury, return to work status) and health service provided (e.g. physiotherapy, orthopaedic surgery). More detail about the NSW Workers’ Compensation system can be found elsewhere [18].

Patients referred to see a physiotherapist from their general practitioner following a work-place injury initially have eight sessions funded by NSW Workers’ Compensation. If a patient requires additional treatment after the eighth session, the treating physiotherapist must justify the need for ongoing treatment in writing and submit this to SIRA. If approved by SIRA, NSW Workers’ Compensation will fund eight more sessions. This process continues until a patient is discharged by the physiotherapist or their condition has plateaued.

### Design and participants

We used data collected by SIRA to identify a cohort of people who received elective lumbar spine surgery (fusion and/or decompression) funded by NSW Workers’ Compensation between 2010 and 2018. Fusion surgery included any case of lumbar fusion with or without decompression; decompression surgery included any decompression (e.g. laminectomy, discectomy) without fusion. Data linkage was used to capture physiotherapy billing codes used before surgery (from date of initial injury to surgery for the same claim). Physiotherapy billing codes covered treatment provided in the clinic (e.g. initial and standard consultation), treatment provided at people’s homes and other activities such as case conference, report writing, and activity assessment (Additional file 1). People were excluded for the following reasons: < 18 years old at the time of surgery, had cervical or thoracic surgery, disc replacement surgery (due to a small number of cases), surgery due to fracture or dislocation, a traumatic brain injury or more than one active workers’ compensation claim, no eligible surgery codes were available, and cost data was missing. We also excluded people with a date of injury prior to 2010 because we did not have data on physiotherapy utilisation prior to 2010 and wanted to capture the entire continuum from injury to physiotherapy to surgery.

### Time from initiation of physiotherapy to surgery, number of physiotherapy sessions, cost of physiotherapy and time from injury to initiation of physiotherapy

Using physiotherapy billing codes, we identified whether a person received any physiotherapy (or none) as well as the time from initiation of physiotherapy to first lumbar spine surgery (fusion vs. decompression).

We categorised this into: ‘< 6 weeks’, ‘6 weeks to <6 months’, ‘6 months to <1 year’, ‘1 year to <2 years’, ‘≥2 years’, vs. ‘no physiotherapy’. People who had a non-treatment billing item as their first and only physiotherapy service provided (i.e. PTA012 and PTA014) were considered to have ‘no physiotherapy’. The above categories were chosen because some guidelines for LBP recommend non-operative management for at least 4–6 weeks before lumbar spine surgery [19–22], while others recommend at least 2 years [23–25]. The number of physiotherapy sessions people received following their workplace injury and before lumbar fusion or lumbar decompression surgery was categorised as: ‘0 sessions’, ‘1–8 sessions’, ‘9–24 sessions’, ‘25–50 sessions’ vs. ‘> 50 sessions’. We chose these categories because NSW Workers’ Compensation approves physiotherapy sessions in blocks of eight. Cost of physiotherapy per claim – reported in Australian Dollars (AUD) – was calculated by summing the cost of each eligible physiotherapy session provided to that patient before their first lumbar spine surgery. Time from injury date to initiation of physiotherapy was categorised as: ‘< 1 week’, ‘1 week to <6 weeks’, ‘≥6 weeks’, vs. ‘no physiotherapy’ before initiating physiotherapy. These categories were chosen to capture the proportion of people who received physiotherapy within 1 week (considered ‘early physiotherapy’ in other papers [26]) and from ≥6 weeks, given most cases of LBP substantially improve within 6 weeks [27].

### Statistical analysis

We used descriptive statistics to summarise – by type of surgery (fusion vs. decompression) – the time (months) from initiation of physiotherapy to surgery [median and interquartile range (IQRs), and counts and percentages], number of physiotherapy sessions people received before surgery [means and standard deviations (SD), and counts and percentages], total annual cost of physiotherapy per claim before surgery (means and IQRs), and time (days) from injury date to initiation of physiotherapy (median and IQRs, and counts and percentages). We excluded non-treatment billing codes (i.e. case conference or report writing – PTA012; and travel – PTA014) from analyses exploring the number of physiotherapy sessions and time from initiation of physiotherapy to surgery. We included all billing codes when considering the cost of physiotherapy. We did not use statistical inference testing as we had data for our target population: people receiving physiotherapy before lumbar spine surgery, funded by NSW Workers’ Compensation. Descriptive statistics were generated using STATA statistical software (version 13.1).

## Results

### Study population

**Fig. 1:**
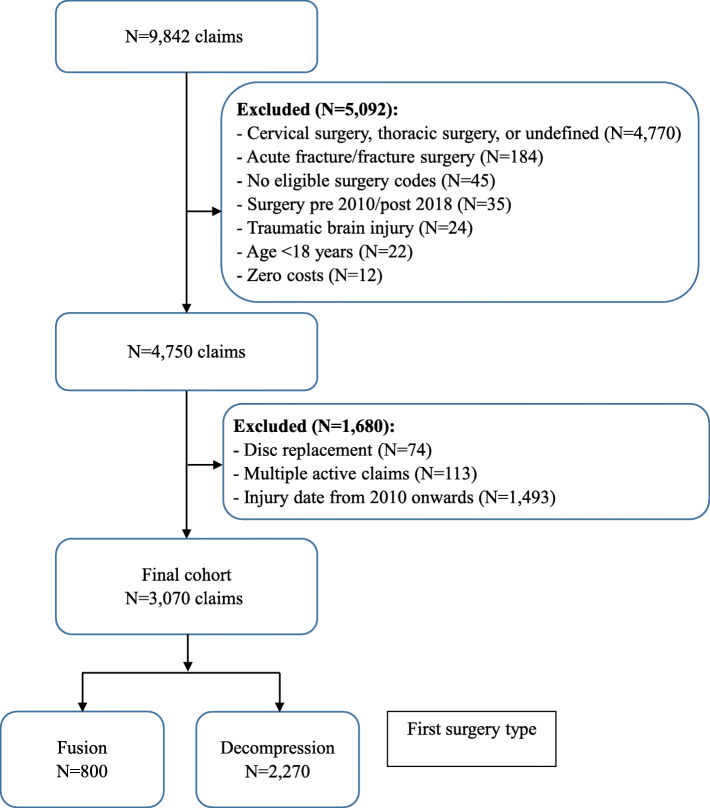
Flow diagram

Data were available for 9842 claims for spine surgery from SIRA between January 1, 2010, and December 31, 2018. After relevant exclusions, our final cohort included 3070 claims/people; 800 people had fusion (with or without decompression) and 2270 had decompression alone as their index lumbar spine surgery (Fig. 1).

### Patient characteristics

The mean age (SD) of people who received fusion was similar to those who received decompression [42.9 (10.4) vs. 41.9 (11.6), respectively], as was the proportion of females (24.6% vs. 22.0%, respectively). The mean (SD) time (months) to first lumbar spine surgery from injury date was higher for people who received fusion compared to decompression [21.6 (17.9) vs. 9.4 (10.6)] (Table 1). The percentage of females was lowest in the subgroups who received no physiotherapy (fusion: 14.6%; decompression: 18.0%) and <  4 weeks of physiotherapy before surgery (fusion: 11.8%; decompression: 20.0%). Mean time to first surgery from injury date increased as the time from first physiotherapy session to first surgery increased. Yet, time to first surgery from injury date was not the lowest among those who had no physiotherapy (Table 1).

**Table 1:** Characteristics of the study population by time from first physiotherapy session to first surgery (fusion vs. decompression)

Demographic and baseline variables	Total sample	No physiotherapy	< 4 weeks	4 weeks to < 6 months	6 months to < 1 year	1 year to < 2 years	≥2 years
**Fusion**							
Mean Age (SD), years	42.9 (10.4)	44.8 (9.7)	45.1 (10.4)	45.1 (11.2)	43.9 (11.0)	41.9 (10.1)	40.9 (9.7)
Gender (Female)	197 (24.6%)	18 (14.6%)	2 (11.8%)	25 (24.3%)	40 (27.0%)	56 (29.6%)	56 (25.5%)
Mean time to first surgery from injury date (SD), months	21.6 (17.9)	14.3 (17.0)	6.8 (6.7)	6.5 (4.5)	11.0 (4.1)	19.1 (5.9)	43.3 (15.9)
n	800	123	17	103	148	189	220
**Decompression**							
Mean Age (SD), years	41.9 (11.6)	43.3 (11.7)	42.5 (11.2)	41.0 (11.4)	41.6 (12.1)	41.8 (11.9)	41.1 (10.6)
Gender (Female)	500 (22.0%)	116 (18.0%)	34 (20.0%)	147 (20.4%)	112 (28.4%)	50 (23.5%)	41 (32.0%)
Mean time to first surgery from injury date (SD), months	9.4 (10.6)	6.1 (8.1)	3.2 (3.6)	5.3 (4.4)	10.2 (5.2)	18.3 (4.9)	39.1 (14.6)
n	2270	644	170	721	394	213	128

*n* Number of people; *SD* Standard deviation

### Time from initiation of physiotherapy to surgery, number of physiotherapy sessions, cost of physiotherapy and time from injury to initiation of physiotherapy

There were 677 (84.6%) people who received at least one physiotherapy session before fusion and 1626 (71.6%) who received at least one physiotherapy session before decompression surgery. The median (IQR) time (months) from first physiotherapy session to surgery was greater for those who received fusion compared to decompression [15 (4 to 26) vs. 5 (3 to 11), respectively]. Among those who received fusion, most people received physiotherapy for 1 year to < 2 years (23.6%) and ≥ 2 years (27.5%) before surgery; one in 50 people (2.1%) received physiotherapy for < 4 weeks before surgery. Among those who received decompression, most people received physiotherapy for 4 weeks to < 6 months (31.8%) or 6 months to < 1 year (17.4%) before surgery; 5.6% received physiotherapy for ≥2 years and 7.5% received physiotherapy for < 4 weeks (Table 2).

**Table 2:** Time from first physiotherapy session to first surgery, number of physiotherapy sessions and cost of physiotherapy before lumbar spine surgery, and time from injury date to first physiotherapy session, by type of surgery

Time from first physiotherapy session to first surgery	Fusion	Decompression
Median (IQR) months^a^	15 (8 to 29)	5 (3 to 11)
Max*	101	92
N	678	1627
	**Fusion**	**Decompression**
	**N (%)**	**N (%)**
No physiotherapy^b^	123 (15.4%)	644 (28.4%)
< 4 weeks	17 (2.1%)	170 (7.5%)
4 weeks to <6 months	103 (12.9%)	721 (31.8%)
6 months to <1 year	148 (18.5%)	394 (17.4%)
1 year to <2 years	189 (23.6%)	213 (9.4%)
≥2 years	220 (27.5%)	128 (5.6%)
N	800	2270
**Number of sessions from first physiotherapy session to first surgery**^**c**^	**Fusion**	**Decompression**
Mean (SD) sessions	28 (35)	16 (21)
Max*	387	172
N	800	2270
	**Fusion**	**Decompression**
	**N (%)**	**N (%)**
0 sessions	124 (15.5%)	645 (28.4%)
1–8 sessions	126 (15.8%)	506 (22.3%)
9–24 sessions	221 (27.6%)	571 (25.2%)
25–50 sessions	184 (23.0%)	394 (17.4%)
> 50 sessions	145 (18.1%)	154 (6.8%)
N	800	2270
**Cost of physiotherapy**^**d**^	**Fusion**	**Decompression**
Mean (IQR) cost	$2357 ($453 to $2947)	$1265 ($0 to $1808)
Max*	$53,249	$19,578
N	800	2270
**Time from injury date to first physiotherapy session**	**Fusion**	**Decompression**
Median (IQR) days^a^	23 (9 to 66)	19 (7 to 53)
Max*	1382	2064
N	678	1627
	**Fusion**	**Decompression**
	**N (%)**	**N (%)**
No physiotherapy^b^	123 (15.4%)	644 (28.4%)
<1 week	124 (15.5%)	375 (16.5%)
1 week to <6 weeks	320 (40.0%)	767 (33.8%)
≥6 weeks	233 (29.1%)	484 (21.3%)
N	800	2270

*n* Number of people; *IQR* Interquartile range; *SD* Standard deviation.

^a^excluding people who had ‘no physiotherapy’; ^b^people who had a non-treatment billing item as their first and only physiotherapy service provided (i.e. PTA012 and PTA014) were considered to have ‘no physiotherapy’; ^c^excludes non-treatment billing items (PTA012 and PTA014); ^d^includes non-treatment billing items (PTA012 and PTA014)

*: minimum value was zero for all analyses

The mean (SD) number of physiotherapy sessions people received from first physiotherapy session to first surgery was higher among those who received fusion compared to decompression [28 (35) vs. 16 [21], respectively]. Among people who received fusion, most received 9–24 (27.6%) or 25–50 (23.0%) physiotherapy sessions before surgery; 18.1% received > 50 sessions. Among people who received decompression, most received 1–8 (22.3%) and 9–24 (25.2%) physiotherapy sessions before surgery; 6.8% received > 50 sessions (Table 2).

The mean (IQR) total cost of physiotherapy before surgery was $2357 ($453 to $2947) for those who received fusion and $1265 ($0 to $1808) for those who received decompression. The median (IQR) time (days) from injury date to first physiotherapy session was greater for those who received fusion compared to decompression [23 (9 to 66) vs. 19 (7 to 53), respectively]. Among people who received fusion, most received physiotherapy from within 1 to < 6 weeks (40.0%) or ≥ 6 weeks (29.1%) of the injury date; 15.4% received physiotherapy within 1 week of injury. Similarly, among people who received decompression, most received physiotherapy from 1 to < 6 weeks (33.8%) and ≥ 6 weeks (21.3%) from injury date; 16.5% received physiotherapy within 1 week of injury (Table 2).

## Discussion

### Summary of main findings

There is variation in physiotherapy utilisation before lumbar spine surgery for people funded by NSW Workers’ Compensation. Some people may not be receiving an adequate trial of physiotherapy before surgery, particularly before decompression surgery. For example, 15.4% of people had no physiotherapy and 15.8% had 1–8 physiotherapy sessions before fusion, while 28.4% had no physiotherapy and 22.3% had 1–8 physiotherapy sessions before decompression. Some people, on the other hand, may be receiving an excessive amount of physiotherapy before surgery, particularly before fusion. For example, one in four people who had fusion received physiotherapy for ≥2 years before surgery, compared to one in 20 people who had decompression. People who had fusion were also more likely to receive 25–50 (23.0%) and > 50 (18.1%) physiotherapy sessions before surgery compared to those who had decompression (17.4 and 6.8%, respectively). In the absence of high-level evidence informing what are appropriate doses of physiotherapy, it is unclear whether over- or under-servicing is occurring.

### Strengths and weaknesses of this study

This is the first study to investigate how much physiotherapy people receive – and for how long – from initiation of physiotherapy to lumbar fusion and lumbar decompression surgery, in Australia, and in a setting involving workers’ compensation. The main strength of this study is that we had data to capture the entire continuum from injury to physiotherapy to surgery. No other study, to the best of our knowledge, has reported this type of data for people undergoing lumbar spine surgery. Other strengths include 9 years of data, a large sample size, and available data for nearly the entire sample of people who had lumbar spine surgery funded by NSW Workers’ Compensation between 2010 and 2018.

The main limitation is that we did not have data on what treatment physiotherapists provided nor data on patient-reported outcomes (e.g. pain, disability). We also did not have data on indications for surgery. This includes the percentage of people with progressive neurological symptoms and the percentage of people who ‘failed’ non-operative management for LBP. Such information could have been used to better understand what health professionals and people consider ‘failed’ conservative management, and whether this has changed over time. We also acknowledge that our findings may not be generalisable to cohorts who did not experience a work-place back injury (or develop LBP at work) and who are not funded by workers’ compensation. For example, people who have their treatment paid for through workers’ compensation might be more willing to try physiotherapy for a prolonged period before surgery compared to those who have to pay out-of-pocket. Further, people who experience a work-place injury may have negative perceptions about their work-place (‘blue flags’) or an objectively harmful work-place (‘black flags’) [28]. These factors may negatively influence recovery and make the management of patients funded by workers’ compensation more complex.

### Meaning of this study

Variation in physiotherapy utilisation before lumbar spine surgery may suggest some people do not receive an adequate trial of physiotherapy before surgery, while others receive an excessive amount of physiotherapy. Nevertheless, it is important to view this interpretation with caution. Since there are no robust data on optimal physiotherapy dosage (and content) before surgery, and we did not have data on patients’ clinical presentation, we do not know whether variation in physiotherapy utilisation was clinically justified or not. For example, people may have only received a small amount of physiotherapy before surgery because they had severe or progressive neurological symptoms.

Overall, use of physiotherapy before surgery was less common and intensive among people who had decompression compared to those who had fusion. Compared to people who had spinal fusion surgery, those who had decompression were more likely to have no physiotherapy before surgery (28.4% vs. 15.4%), received physiotherapy for less time before surgery (median: 5 vs. 15 months) and had fewer physiotherapy sessions before surgery (mean: 16 vs. 28 sessions). Health professionals and people considering decompression may have been less willing to trial an extended period of non-operative management before surgery because decompression is a less invasive procedure than fusion (possibly with fewer risks) and is conditionally recommended in some guidelines [8]. For fusion surgery, it is possible that many people had excessive physiotherapy without benefit, as 27.5% had over 2 years of physiotherapy followed by spine fusion.

All people in our sample had lumbar spine surgery, so our data cannot be used to conclude physiotherapy does not improve outcomes for people considering surgery. Evidence-based clinical practice guidelines for LBP support several physiotherapy-delivered treatments, such as “stay active” advice, reassurance, exercise and manual therapy. Physiotherapy is also a ‘relatively’ inexpensive treatment option. The cost of lumbar spine surgery in Australia is ~$53,000 per operation [29]. Physiotherapy cost much less in our sample ($2357 before fusion and $1265 before decompression per patient), although physiotherapy costs in this cohort were in addition to surgical costs because all participants had surgery.

### Comparison to existing literature

Only one study has investigated the number of physiotherapy sessions people received before lumbar spine surgery; none have explored the issue of duration of physiotherapy. A study of 30,709 people in the USA who had lumbar discectomy for disc herniation from 2004 to 2006 found people, on average, had 6 physiotherapy sessions in the 90 days before surgery [16]. This was less than our study: 28 physiotherapy sessions before fusion and 16 before decompression, but this difference is likely explained by the fact that we captured all physiotherapy between injury and surgery, with no limits to the data collection period before surgery.

Four studies – three from the USA – have investigated the use and costs of non-operative treatment before lumbar spine surgery; none focus on compensable people. The above-mentioned study found non-operative management cost people $3445 in the 90 days before surgery – physiotherapy treatment accounted for 11% of these costs ($379 per patient) [16]. A study of 27,877 people in the USA who had fusion for lumbar stenosis or spondylolisthesis from 2007 to 2015 found 40% received physiotherapy or occupational therapy in the 2 years prior to surgery (costing $83 per patient in 2015) [13]. A similar study of 13,106 people in the USA who had lumbar microdiscectomy for disc herniation from 2007 to 2017 found 16% of people received physiotherapy or occupational therapy in the 3 months prior to surgery (costing $79 per patient). The same study also found geographical variation in physiotherapy use and costs (South: 15% of people, $74 per patient; Northeast: 16%, $66; West: 17%, $78; and Midwest: 20%, $89) [14]. A survey of 229 people with LBP referred for elective lumbar spine surgery in Ontario (Canada) found half of the people had received physiotherapy in the 2 years prior to having a consult for surgery [15]. Use and cost of physiotherapy before surgery was higher in our sample (fusion: 85% received physiotherapy, costing $2357 per patient; decompression: 72% received physiotherapy, costing $1265 per patient) compared to the above studies. We captured all physiotherapy between injury and surgery, with no restriction to the data collection period before surgery, which may explain these differences.

We did not have data on utilisation and costs of other non-operative treatments before lumbar spine surgery. The above studies shed some light on this topic. One study from the USA found that non-operative treatment costs (total costs in the 90 days before lumbar discectomy for disc herniation: $3445 per patient) were accounted for by spinal injections (32% of total costs), diagnostic imaging (31%), outpatient visits (13%), physiotherapy (11%), chiropractic manipulation (2%), preoperative studies (0.8%), medications (0.5%), and other charges [16]. Another study in the USA found that the total cost of non-operative treatment in the 2 years prior to fusion surgery ($2204 per patient in 2015) was accounted for by lumbar epidural steroid injections ($803 per patient), emergency department visits ($737), chiropractic treatment ($224), non-steroidal anti-inflammatory drugs (NSAIDs; $147), opioids ($142), physiotherapy or occupational therapy ($83), and muscle relaxants ($68) [13]. Similar patterns of utilisation and costs of non-operative treatments were found in the 3 months period before lumbar microdiscectomy for disc herniation in another study in the USA [14]: lumbar epidural steroid injections (received by 29% of people, $887 per patient over 3 months), chiropractic treatment (12%, $136 per patient), NSAIDS (24%, $53 per patient), opioids (60%, $41 per patient), and physiotherapy or occupational therapy (16%, $79 per patient).

### Unanswered questions and future research

This is the first study to explore physiotherapy utilisation and costs before lumbar spine surgery in Australia, in a setting involving Workers’ Compensation and considering the entire continuum from injury to physiotherapy to surgery. It was also the first, to the best of our knowledge, that explored physiotherapy utilisation and costs before lumbar decompression surgery. More research is needed to explore whether our findings vary across cohorts of compensable people in different countries.

Our data did not include specific details of the physiotherapy-delivered treatments people received before surgery. Inadequate reporting of specific treatments provided by physiotherapists is a common barrier to understanding what care patients receive across various settings (e.g. private sector, paid by health insurers, in hospitals, workers’ compensation cohorts). A 2019 systematic review of 94 studies across 19 countries investigated physiotherapists’ treatment choices for various musculoskeletal conditions through surveys of physiotherapists and audits of clinical notes (including 48 studies of LBP) [30]. Unfortunately, none of the included studies were in cohorts that eventually went on to have lumbar spine surgery (to our knowledge). The previously mentioned cross-sectional survey (*n* = 229 people in Canada) may be the only study to provides insight into what treatment physiotherapists provide to people referred for elective lumbar spine surgery [15]. Commonly utilised physiotherapy treatments in this context include exercise (57%), massage (27%), other adjunct modalities (29%; e.g. acupuncture, electromyography biofeedback, relaxation). Future research, for example involving audits of physiotherapists’ clinical notes, is needed to better understand what physiotherapy-delivered treatments people try before deciding to have lumbar spine surgery in settings involving workers’ compensation, as well as in other settings and for other conditions. Future research should also determine “how much” non-operative management is adequate before lumbar spine surgery, considering time from initiating non-operative treatment to surgery and volume of non-operative treatment.

## Conclusion

There is variation in physiotherapy utilisation before lumbar spine surgery for people funded by NSW Workers’ Compensation. Some people may not be receiving an adequate trial of physiotherapy before surgery, particularly before decompression surgery. Others may be receiving an excessive amount of physiotherapy before surgery, particularly before fusion. Overall, people who had decompression received physiotherapy for less time and had fewer physiotherapy sessions before surgery compared to those who had fusion.

## Supplementary Information

**Additional file 1.**

## Data Availability

The datasets used and/or analysed during the current study are available from the corresponding author on reasonable request.

## Abbreviations

AUD: Australian dollars
IQR: Interquartile range
LBP: Low back pain
NSAIDs: Non-steroidal anti-inflammatory drugs
NSW: New South Wales
SD: Standard deviation
SIRA: State insurance regulatory authority
USA: United States of America
