# Supplementary material for: Physiotherapy utilisation and costs before lumbar spine surgery: a retrospective analysis of workers compensation claims in Australia
Source: BMC Musculoskelet Disord. 2021 Mar 6;22:248. doi: 10.1186/s12891-021-04129-4 (PMC7937209; doi:10.1186/s12891-021-04129-4)
Supplement: Supplementary file 1 — Additional file 1. [file 12891_2021_4129_MOESM1_ESM.docx]

**Physiotherapy utilisation and costs before lumbar spine surgery: a retrospective analysis of workers compensation claims in Australia**

Joshua R. Zadro^1, 2*^, Adriane M. Lewin^3^, Priti Kharel^1, 2^, Justine Naylor^4^, Christopher G. Maher^1, 2^, Ian A. Harris^1, 2, 3^.

^1^Sydney School of Public Health, Faculty of Medicine and Health, The University of Sydney, Sydney, NSW, Australia.

^2^Institute for Musculoskeletal Health, Sydney Local Health District, Sydney, NSW, Australia.

^3^Ingham Institute for Applied Medical Research, South Western Sydney Clinical School, University of New South Wales, Sydney, NSW, Australia.

^4^Whitlam Orthopaedic Research Centre, Orthopaedic Department, Liverpool Hospital, Sydney, NSW, Australia.

*Correspondence to Joshua Robert Zadro: [joshua.zadro@sydney.edu.au](mailto:joshua.zadro@sydney.edu.au). Level 10 North, King George V Building, Royal Prince Alfred Hospital, PO Box M179, Missenden Road, Camperdown, NSW 2050, Australia.

**Appendices**

| Appendix A. Description of billing items | |
| --- | --- |
| **Item number** | **Description** |
| PTA001 | Initial consultation and treatment |
| PTA002 | Standard consultation and treatment |
| PTA003 | Initial consultation and treatment of two distinct areas |
| PTA004 | Standard consultation and treatment of two distinct areas |
| PTA005 | Complex treatment |
| PTA006 | Group / class Intervention (rate per participant) |
| PTA007 | Initial consultation and treatment |
| PTA008 | Standard consultation and treatment |
| PTA009 | Initial consultation and treatment of two distinct areas |
| PTA010 | Standard consultation and treatment of two distinct areas |
| PTA011 | Complex treatment |
| PTA012 | Case conference (p/hour), Report Writing (p/hour - max) |
| PTA013 | Activity assessment, consultation and treatment |
| PTA014 | Travel |
